# Polyether-Ether-Ketone (PEEK) and Its 3D-Printed Quantitate Assessment in Cranial Reconstruction

**DOI:** 10.3390/jfb14080429

**Published:** 2023-08-17

**Authors:** Khaja Moiduddin, Syed Hammad Mian, Sherif Mohammed Elseufy, Hisham Alkhalefah, Sundar Ramalingam, Abdul Sayeed

**Affiliations:** 1 Advanced Manufacturing Institute, King Saud University, Riyadh 11421, Saudi Arabia; 2 Department of Oral and Maxillofacial Surgery, College of Dentistry and Dental University Hospital, King Saud University Medical City, Riyadh 11545, Saudi Arabia; 3 Department of Mechanical Engineering, College of Engineering, King Saud University, Riyadh 11421, Saudi Arabia

**Keywords:** cranial defects, polyether-ether-ketone, porous implants, 3D printing, biomechanical analysis, fitting analysis

## Abstract

Three-dimensional (3D) printing, medical imaging, and implant design have all advanced significantly in recent years, and these developments may change how modern craniomaxillofacial surgeons use patient data to create tailored treatments. Polyether-ether-ketone (PEEK) is often seen as an attractive option over metal biomaterials in medical uses, but a solid PEEK implant often leads to poor osseointegration and clinical failure. Therefore, the objective of this study is to demonstrate the quantitative assessment of a custom porous PEEK implant for cranial reconstruction and to evaluate its fitting accuracy. The research proposes an efficient process for designing, fabricating, simulating, and inspecting a customized porous PEEK implant. In this study, a CT scan is utilized in conjunction with a mirrored reconstruction technique to produce a skull implant. In order to foster cell proliferation, the implant is modified into a porous structure. The implant’s strength and stability are examined using finite element analysis. Fused filament fabrication (FFF) is utilized to fabricate the porous PEEK implants, and 3D scanning is used to test its fitting accuracy. The results of the biomechanical analysis indicate that the highest stress observed was approximately 61.92 MPa, which is comparatively low when compared with the yield strength and tensile strength of the material. The implant fitting analysis demonstrates that the implant’s variance from the normal skull is less than 0.4436 mm, which is rather low given the delicate anatomy of the area. The results of the study demonstrate the implant’s endurance while also increasing the patient’s cosmetic value.

## 1. Introduction

Cranioplasty is one of the earliest forms of neurosurgery, and it consists of surgically correcting a cranial defect, usually in a delayed fashion, to reduce the danger of graft infections [1]. Cranial abnormalities can be caused by a variety of factors, including trauma, decompression surgeries, tumors, infections, congenital damage, or iatrogenesis [2]. Cranial defects cause functional, cosmetic, and psychological changes that have a substantial impact on a patient’s quality of life. There are various methods for the reconstruction of cranial defects, and it is crucial to consider a number of variables, including the biomaterial to be used, the costs, the type of surgery to be done, its associated morbidity, and the implant stability over time [3,4]. There are direct and indirect methods for fabricating cranial implants. The indirect method consists of creating a realistic model of the defective region, which acts as a template for manufacturing through casting or other molding techniques. The traditional methods are associated with an increase in surgical time and frequently yield unsatisfactory cosmetic results. The direct method consists of the integration of computer-aided design (CAD) and direct digital additive manufacturing of extremely precise and personalized implants based on Computed Tomography (CT) scan data.

As per the literature studies, the ideal material for cranioplasty should be radiolucent, immune to infections, thermally inert, biomechanically robust, flexible, well-fitting, inexpensive, and able to encourage tissue growth on it [5]. Traditional bioimplants composed of titanium and cobalt–chromium alloys have excellent mechanical properties, high corrosion resistance, and ductility. However, in spite of all these benefits, these metal biomaterials have a certain shortcoming, namely the stress-shielding effect and inadequate compliance with modern imaging technologies [6]. The modulus of elasticity of titanium (114 GPa) is more than five times that of human bone (~20 GPa), leading to weakening of the surrounding bone, bone resorption, and implant failure over time [7]. Contrarily, Polyether ether ketone (PEEK) has taken the lead position in polymer-based orthopedic implants due to its advantageous mechanical qualities. 

PEEK (Polyether-ether-ketone) is a thermoplastic polymeric material and a dominant member of the Polymaryletherketone (PAEK) family. The FDA (U.S. Food and Drug Administration) authorized PEEK as an implantable biomaterial in the early 1990s [8]. PEEK is an anti-corrosion and natural radiolucency biomaterial and its mechanical properties match that of natural bone thus making it an excellent candidate for medical prostheses [9]. Since the 1990s, PEEK has been widely regarded as an attractive alternative to metal biomaterials in medical applications such as orthopedics and craniomaxillofacial surgeries [10,11,12]. The traditional technique of producing PEEK implants is through mechanical processing by cutting the block of PEEK mold which is an expensive process and consumes lots of raw material [13,14]. However, there were also concerns and reports of smooth and bulk PEEK implants, which lead to poor osseointegration potentially leading to clinical failure [15]. Consequently, considerable efforts were made to modify the smooth and flat surface to reinforce the PEEK implants in order to enhance and stimulate the growth of osteoblasts. One such technique is converting the flat and smooth surface of PEEK into a porous surface. The long-term durability and the stability of the implant are influenced by how effectively the bond is between the bone and implant surface. Several studies have demonstrated the cell proliferation increase in porous implants over flat surfaces [16,17].

In their study, F. Brennan Torstrick et al. [18] evaluated the bone–implant interface and cell in-growth in smooth PEEK, porous PEEK, and titanium-coated PEEK using in vivo and in vitro analysis. The porous PEEK implant exhibited enhanced osseointegration and cellular proliferation when compared with smooth and Ti-coated PEEK. Similar investigations of titanium and non-PEEK biomaterials revealed that porous surfaces provide superior osseointegration compared with smooth surfaces [19,20]. The introduction of porous implants has two main benefits; on the one hand, it can avoid the stress shielding effect, and, on the other hand, it can provide the growth space for cells, promoting the cell tissue and osseointegration. There are several porous structures that coincide with the bone morphology but, among them, the diamond porous structure has received increased attention due to its high bone ingrowth permeability and bone tissue regeneration [21].

Several methods, including sulfonation, porogen templating, and melt extrusion, have been used to create porous patterns on the PEEK surface [22,23], although, these traditional methods produce a PEEK scaffold (porous structure) but not in a controlled environment. They have limitations in producing free-form geometries and process contamination is also a major factor [24]. In addition, there have been reports of missing scaffold interconnectivity, residual impurities, and dead spaces [25] when producing scaffolds through traditional methods. With the recent introduction of additive manufacturing, scaffolds can be produced in a controlled environment with an optimal pore size and porosity, which is most suitable for cell migration and proliferation. 

Three dimensional-printed PEEK would have a favorable effect on orthodontics as a whole by lowering production costs and allowing for the creation of prostheses that are more suited to individual patients [26,27]. PEEK can be printed utilizing two distinct 3D-printing methods, Selective Laser Sintering (SLS) and Fused Filament Fabrication (FFF). SLS is a method in which a laser or electron beam uses heat to carefully fuse together the powder to make it a solid structure whereas, in FFF printing technology, the filament is loaded into the printing system, usually with the help of a feeder motor. The material is then heated to a semi-liquid state, extruded through a nozzle, and printed layer by layer on a bed until the entire object is formed. SLS printers can achieve a resolution of 50–100 m, but they are prohibitively expensive compared with FDM printers. Consequently, FDM printers are extensively used despite their higher resolution of 100–150 m [28]. In the past decade, 3D-printed PEEK has been a topic of interest for several researchers in numerous industries. Han et al. [29] studied the three-dimensionally printed polyetheretherketone (PEEK) implants for their surface roughness, wettability, cell adhesion, metabolic activity, and proliferation. In their study, Oladapo et al. [30] explained the ideal routes to boost the 3D printing and scientific mechanism of PEEK and its composites. In their study, Saini et al. [31] demonstrated the use of PEEK spinal fusion cages produced through the Funmat HT 3D printer. 

Although there have been a few publications on the extrusion of polymer porous structures [32], the study of PEEK porous structures using AM is still limited due to its very high melting temperature (420 °C), in comparison with the melting temperature of engineering polymer materials such as acrylonitrile butadiene styrene ABS and (polylactic acid) PLA (200 °C). In addition, PEEK is also prone to warping and incomplete crystallization, which hampers the part’s mechanical properties [14]. Hence, critical FFF processing parameters, such as the nozzle, chamber, print speed, and bedplate temperature, should be optimized to help with better layer fusion and provide aesthetic results. The fabrication of the PEEK scaffolds using Fused Filament Fabrication provides various benefits including reduced material wastage, improved cost-effectiveness, faster production, and improved patient specificity [33]. 

Previous research has shown that the optimal porosity for cell growth and nutrient proliferation is between 40 and 70% [34]. Numerous studies have demonstrated that the utilization of porous structures, featuring pore sizes spanning from 100 to 1200 μm and porosity (30–80%), can effectively facilitate the process of osseointegration between bone analogs and healthy stumps [35,36,37,38]. In their study, Naoya et al. [38] demonstrated that a diamond lattice structure with a pore size of 600 µm and a porosity of 65% is ideal for rapid bone ingrowth. In their study, Fei Liu et al. [39] demonstrated that a diamond lattice structure with an interconnected porosity of 81–97% is good for tissue ingrowth and vascularization. In their study, Ashkan Farazin et al. [40] illustrated that a diamond scaffold with a porosity of 60 to 70% demonstrated improved cell viability and bone ingrowth. Naghavi et al. [41] analyzed diamond scaffolds with a pore size of 900 to 1500 µm and found that the scaffolds within 1400 µm were within the acceptable limit of cortical bone stiffness. Goto et al. [42] studied two alternative implant designs, one with a smooth surface and the other with uniform pores ranging from 800 to 1400 µm, and found that lattice-shaped interconnected implants are a superior alternative for medical application. Based on the literature survey, porosities ranging from 25% to 90% and pore sizes ranging from 100 to 1500 µm were discovered to be the most commonly employed in the design of porous implants. During the design of a porous implant, there is frequently a trade-off between porosity percentage and mechanical strength. Through osseointegration, a porous implant’s mechanical interlock strength can be increased; however, a design with a porosity of more than 80% leads towards a loss in both strength and bone ingrowth [43]. Although there have been several research studies on porous implants, the optimization and performance of porous structures still need attention, especially the pore structure, size, direction, and porosity [44]. Li et al. [45] demonstrated that the elastic modulus is 25.9 GPa when the pore size is 0.65 mm, and 14.5 GPa when the pore size is 0.5 mm, which is comparable to the elastic modulus of adult cortical bone. Karaman et al. [46] conducted compression tests on different design models with 50%, 60%, 70%, 80%, and 90% porosities and found that the structural strength decreases with increasing porosity. The mechanical properties of porous materials, like their elastic modulus and yield stress, are linked to their porosity. The proper tailoring of pore size, porosity, direction, and transition area height is essential in order for the biomimetic implant design to work.

The diamond lattice has several benefits, including its strut orientation, which resembles that of trabecular bone [40,47]. Diez-Escudero et al. [48] demonstrated that, among all geometries, the diamond structure has the highest interconnectivity levels and the most uniform distribution of pore sizes. Among the various 3D lattice structures, the diamond unit cell is a promising topology structure that resembles bone topology and is commonly used for orthopedic applications [49,50]. The strut orientation of the diamond structure is also convenient in 3D printing [47]. However, it has also been emphasized in the literature that the employment of appropriate size and density is very critical to accomplish the desired properties in any porous structure [51]. 

Another crucial factor to consider while developing an implant is its load-bearing capacity. The effect of pore size and porosity on the physical and mechanical behavior of a lattice structure is essential. It is evident that when the pore size/porosity of a lattice structure grows, more empty space inside the structure is formed, allowing for possible material diffusion. As a result, it is commonly advocated that the porosity percentage of a lattice structure placed in an implant should be equivalent to that of cancellous bone, resulting in a mean relative density of roughly 20% [52]. A quantitative evaluation based on biomechanical paradigms may be useful for implant mechanical stability. Numerical simulations have therefore emerged as a crucial tool in the area of biomechanics, due to their ability to estimate a design’s load-bearing capacity without the need for a prototype and mechanical testing [53,54]. 

In this study, we have designed and fabricated a porous (diamond scaffold) PEEK implant with a 70% porosity and a pore diameter of 1350 µm for cranial reconstruction using FFF and assessed the implant fitting accuracy using a 3D comparison technique. Although there are fewer studies related to customized PEEK implants, there are hardly any studies related to biomechanical implants and the accuracy assessment of a porous (diamond scaffold) PEEK implant and its process workflow from CT scan to surgery. The purpose of this study is to illustrate the applicability of a tailored PEEK porous implant and the evaluation of its fitting accuracy.

## 2. Proposed Methodology

**Figure 1:**
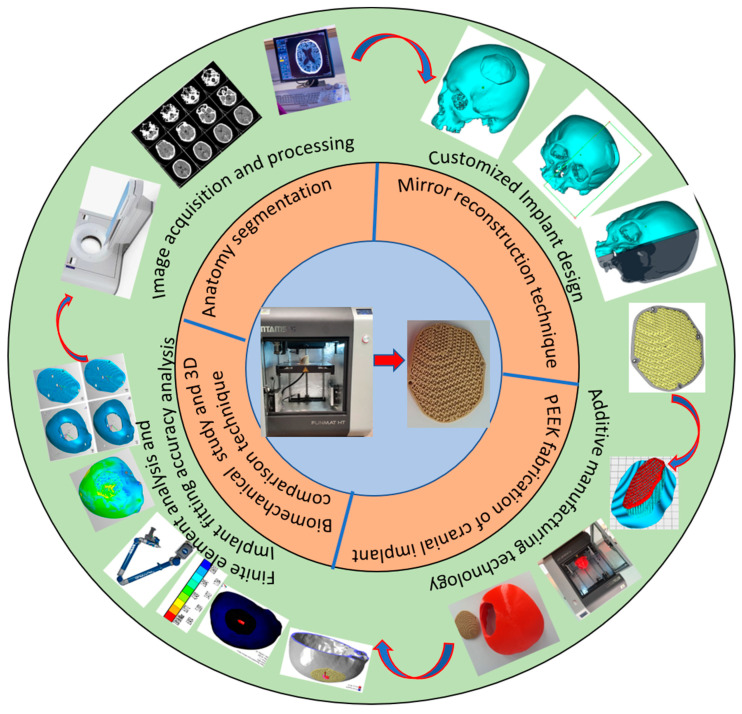
Methodology used for the fabrication of porous PEEK implant for cranial reconstruction.

Figure 1 illustrates the process flow used in the reconstruction of porous PEEK implants for cranial reconstruction. It consists of four stages including image acquisition and processing, mirror reconstruction for the implant design, additive manufacturing of the designed PEEK cranial implant, and finally, the implant biomechanical study and fitting and accuracy analysis.

### 2.1. Image Acquisition and Processing

**Figure 2:**
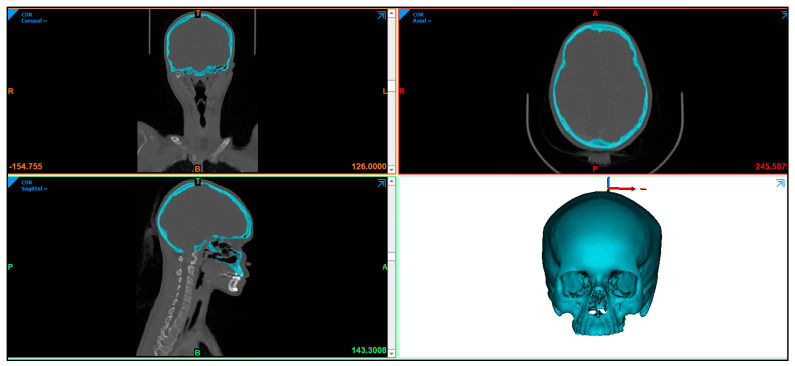
Radiographic image displaying the skull anatomy and the creation of 3D image model.

In this study, a clean skull is used as a reference model to ensure that the designed customized cranial implant is evaluated accurately. The CT scan images are imported into Mimics^R^ 17.0 (Materialise, Leuven, Belgium), a medical modeling software where the images are processed using segmentation and region growing techniques and converted into a 3D Image model, as shown in Figure 2.

**Figure 3:**
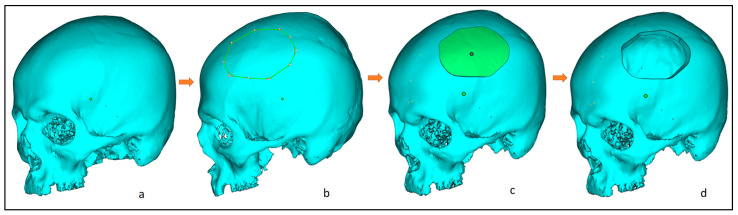
Steps involved in the creation of an experimental segmental defect, from left to right: (**a**) clean skull model; (**b**) marking of the experimental defect; (**c**) after segmentation; and (**d**) resecting the segmental region and obtaining the final segmental skull defect.

The 3D image model (Figure 3a) is imported into 3-Matic (Materialise, Leuven, Belgium) to create an experimental segmental defect. Figure 3 illustrates the process flow for a segmental defect where an experimental segmental defect (Figure 3b) is marked (green lines) on the outer skull surface and resected to generate the segmental defect (Figure 3c,d). The significance of having the healthy skull model and the creation of an experimental segmental defect is to assess the designed cranial implant and compare it with the healthy skull model for the accuracy analysis. All experiments were performed in accordance with the guidelines and approval of the institutional review board committee (Project No. E-22-7235 and approval letter reference number 23/0012/IRB-A).

### 2.2. Customized Implant Design

**Figure 4:**
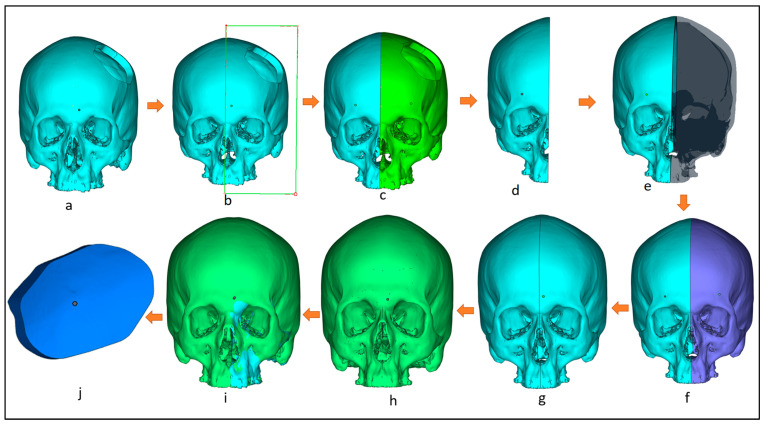
Workflow for the creation of customized implant design involving: (**a**) 3D model with segmental defect; (**b**) creating a center datum plane to resect the skull into equal halves; (**c**) resection the model into healthy and defective regions; (**d**) removing the defective region; (**e**) mirroring the healthy right region; (**f**) obtaining skull mirroring operation; (**g**) performing wrapping and merging operation to remove voids and gaps in between; (**h**) obtaining defect-free 3D model; (**i**) performing boolean subtraction operation between the segmental model and the obtained defect free model; (**j**) obtained bone which as a template for implant design.

The design of the customized cranial implant is based on the defect created on the healthy skull. There are several implant design techniques but, among them, the most commonly used is mirror reconstruction. Figure 4 illustrates the design process for the mirror reconstruction process. In the mirror reconstruction technique, a center datum plane (Figure 4b) is generated on the experimental segmental defect model to resect the skull into two equal symmetrical parts, right and left, known as healthy and defective portions (Figure 4b,c). The defective left portion (green) is removed (Figure 4d) and replaced with a healthy right portion using mirror operation in 3-Matic (Figure 4e,f). Merge operation is performed to join both error-free portions (Figure 4g). The gaps and voids are removed through a wrapping operation to obtain a defect-free 3D model (Figure 4h). Boolean subtraction operation (Figure 4i) is done between the defect-free 3D model (Figure 4h) and the defective model (Figure 4a) to obtain the implant–bone model (Figure 4j), which is saved as an STL file. 

**Figure 5:**
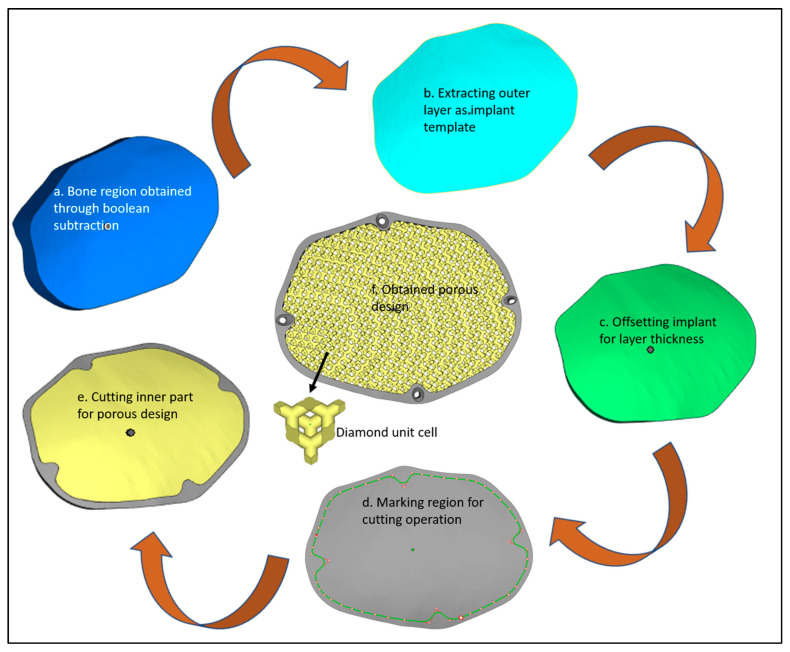
Design steps in the creation of the porous cranial implant.

The obtained implant-bone region (Figure 5a) is imported into Magics (Materialise, Leuven, Belgium) for the designing of the implant. At first, the outer surface of the implant-bone model is extracted (Figure 5b) which acts as an implant template. An offset layer thickness of 4 mm is provided in the model (Figure 5c). Next cutting operation is performed to obtain two regions—outer and inner regions (Figure 5d). The outer region (Figure 5e) is left for the creation of four screw holes whereas the inner portion is transformed into a diamond porous scaffold using a magic structure module (Figure 5f) with 70% porosity and saved as an STL (Standard Tessellation Language) file.

**Figure 6:**
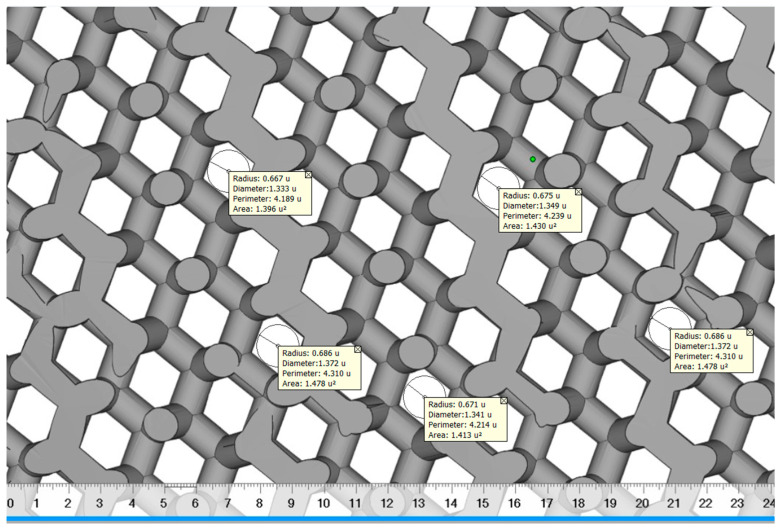
The pore diameter measurement of diamond scaffold using digimizer.

The diamond scaffold pore diameter was measured using digimizer image analyzer software as shown in Figure 6. Digimizer (MedCalc Software Ltd., Ostend, Belgium) is an intuitive precise image measurement tool for analyzing various types of digital images including X-rays, micrographs and images [55]. A total of 5 reading was taken and a mean value was calculated. The diamond scaffold was found to have a mean pore diameter of approximately 1350 μm and a porosity of approximately 70%.

Designed porosity was calculated according to the following equation, where the volume parameters were obtained from STL files using magics 21.0^®^.

(1) $$\mathrm{Porosity}\%=(\frac{V_{1}-V_{2}}{V_{1}})\times 100$$

where *V*_1_ is the volume of the bulk implant found to be 18,719.33 mm^3^ and *V*_2_ is the volume of the diamond scaffold measured as 5571.13 mm^3^.

### 2.3. PEEK Fabrication of Cranial Implant

The STL file is a standard input format for Additive manufacturing. Initially, the designed porous STL file is subjected to correction of errors through Magic (Materialise, Leuven, Belgium) before fabrication. The STL file represents the outer side of the 3D model using a number of triangles connected to each other in a 3D network mesh. The volume of the model is specified by the mesh. This 3D triangular mesh has some common defects such as overlapping triangles, inverted normal, noise shells, and intersecting triangles. Repairing the STL file is important before starting to print using AM technology. 

**Figure 7:**
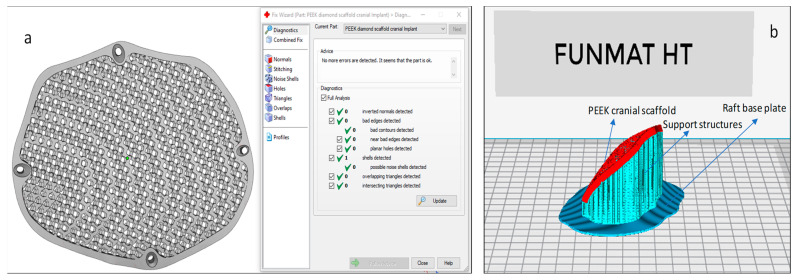
(**a**) Magics and (**b**) Intamsuite software used for STL preparation and support generation.

**Figure 8:**
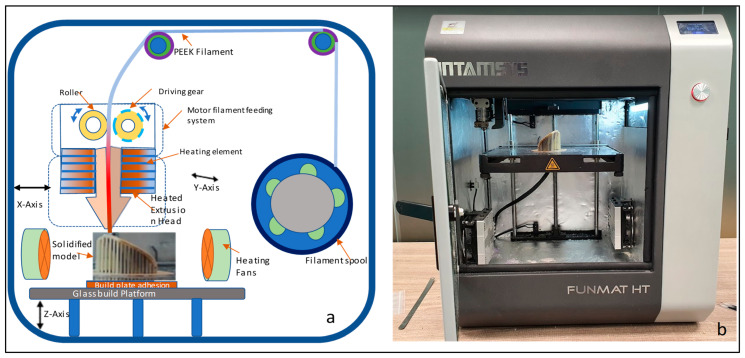
(**a**) Schematic diagram of Fused Filament Fabrication process and (**b**) Funmat HT 3D printer used for the production of PEEK implant.

In this study, Magics 18.0 (Materialise, Leuven, Belgium) is used to repair the porous STL file (Figure 7a). Once the STL file is repaired, INTAMSUITE 3.6.2 a slicing software (Figure 7b) is used to slice the STL file, produce the appropriate supports for overhanging structures, and generate the G-CODE for the print. A raft adhesion-built plate is added to the bottom to ensure a stronger adhesion base and to increase the bed surface area for efficient heat transfer. The Intamsys FUNMAT HT (Intamsys Technology Co., Ltd., Shanghai, China) 3D printer is employed in this study, which works on the FFF principle. The filament (PEEK) of 1.75 mm diameter is fed from a spool via a heated extruder head and deposited on a constructed platform as illustrated in Figure 8.

With computer control, the extruder or print head moves in the X and Y dimensions in accordance with the CAD model to produce the desired shape. Upon completion of each layer, the print head is lowered vertically in the Z direction to start a new layer of material until the print is completed. A closed chamber with a bed temperature of 160 °C, a chamber temperature of 120 °C, and a print temperature of 420 °C is used for printing. The slicing software’s infill parameter is set to 100% to create an entirely solid structure. Table 1 lists the manufacturing process variables used to create the PEEK porous cranial implant.

**Figure 9:**
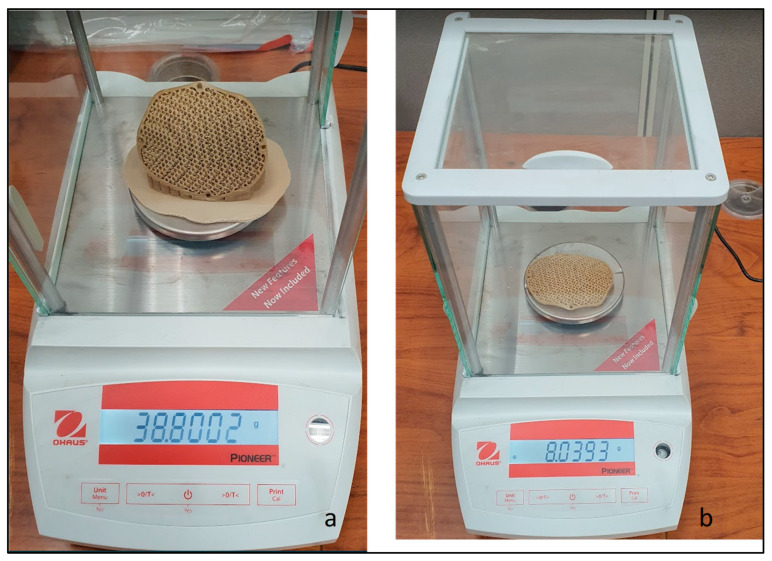
Weight scale reading of PEEK porous implant with (**a**) supports and (**b**) without supports.

**Figure 10:**
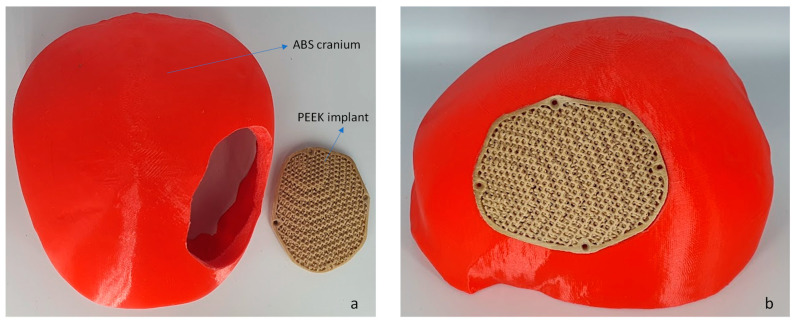
(**a**) The fused filament fabrication of cranium using ABS material and (**b**) PEEK porous implant employed for fitting investigation.

Figure 9 illustrates the printed porous weight of the PEEK implant with supports and without supports. The same INTAMSYS 3D Printer is used to create a skull model (Figure 10) using acrylonitrile butadiene styrene (ABS) material for testing and assessment. The fabrication of a porous PEEK cranial implant took approximately 4 h and 25 min to produce, and it costs about $60 to print. The ABS skull model is evaluated for cranial implant custom fitting and rehearsal evaluation. The porous implant precisely fits on the defective skull region, thus providing good aesthetic performance. To further quantify the implant and skull fitting assessment, a 3D comparison technique is followed whereby state-of-the art Faro arm scanner is used to estimate the deviation and inaccuracy. 

### 2.4. Biomechanical Study

**Figure 11:**
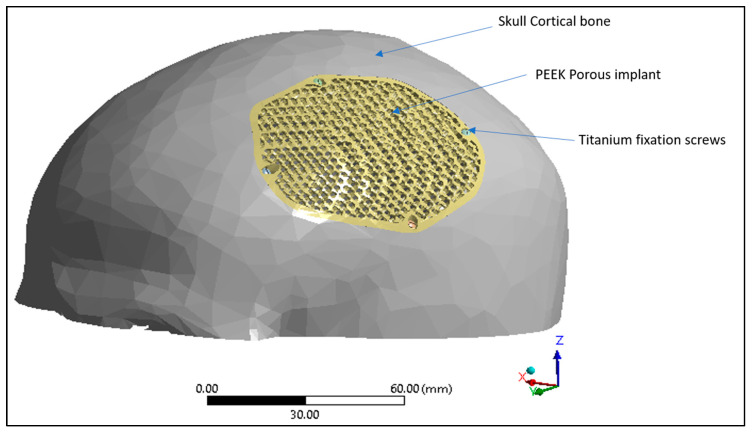
Finite element model displaying skull cortical bone, PEEK porous implant and the titanium fixation screws.

The biomechanical study is the mechanical behavior of the material to ensure that the final product is in conjunction with the designed expectations. It is an important procedure to further improve the design workflow and to ensure patient safety. In order to validate the strength of the designed PEEK porous structure, biomechanical analysis was performed. Sabik et al. [56] in their study, validated numerical and experimental studies of 3D-printed dog bone specimens for the preparation of personalized models in medical applications. Previous studies have also performed biomechanical analysis on the behavior of the material in large cranial defects [57]. Finite element method is an effective method to evaluate the design of the porous implant. The ANSYS software (Version 19.1, Canonsburg, PA, USA) and Hypermesh program (Version 14.0, Altaire Hyper works, Troy, MI, USA) were utilized for pre-processing, post-processing, and execution of the constructed finite element model. The constructed computational model comprises three distinct components, namely the Skull, the PEEK implant, and the fixation screws. The properties of the materials assigned to the FE (Finite element) model are presented in Table 2. Distinct material properties are assigned to various regions of the Finite element model. The cranium is attributed with cortical bone features, whereas the customized cranial implant is endowed with PEEK attributes and four titanium screws are affixed at specific reference points as shown in Figure 11. In this study, the material description is considered isotropic to ensure consistent behavior and to facilitates the application of a linear model [58]. 

**Figure 12:**
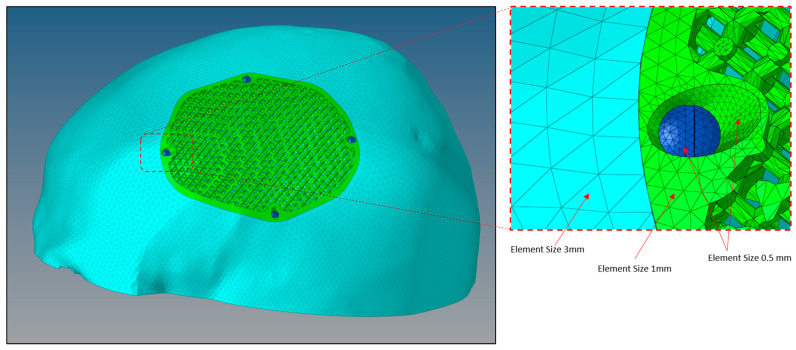
Size of mesh elements in the finite element model of a porous PEEK implant (green) connected to a skull (sky blue) with titanium screws (dark blue) produced through Hypermesh.

The interface between the plate-bone and the bone-screws is designated as bonded [61,62]. The finite element (FE) model of the skull and implant is established using the solid element of the tetra4 type, with the application of Hypermesh. The implant model achieved a fine mesh transition through the implementation of refinement techniques. The mesh dimensions range from 0.5 to 3 mm as shown in Figure 12. To optimize element quality and minimize the mesh distortion, a finer mesh consisting of 814,580 elements and 170,929 nodes was generated for the finite element model as shown in Table 3.

**Figure 13:**
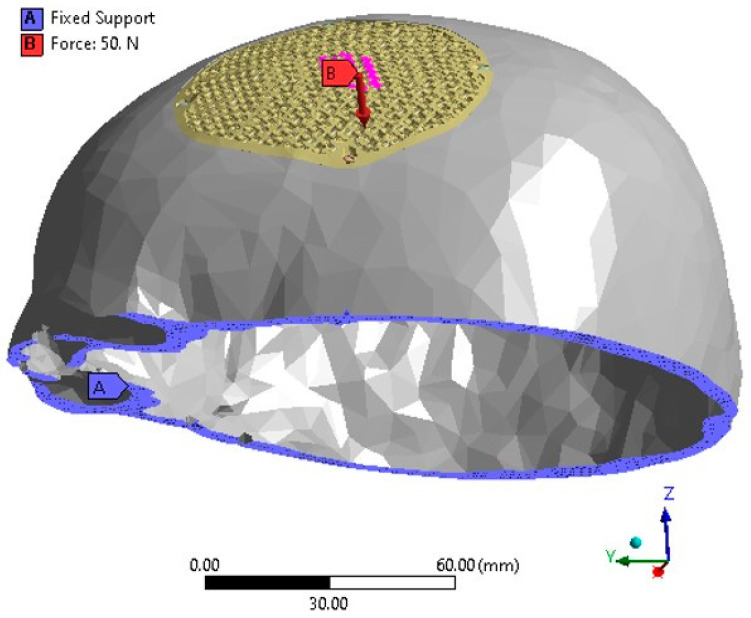
The FEM depicting the loading and boundary conditions.

The finite element model of the PEEK implant is subjected to simulate loading and boundary conditions in order to examine the strength of a porous PEEK design as shown in Figure 13. The base of the cranium is maintained constant by anchoring it at the bottom, and a static force of 50 N is exerted over an area of 200 mm^2^ at the center of the implant [63,64]. The force of 50 N corresponds to the gravitational force of the patient’s head [65]. In accordance with medical professionals’ guidance, the static loading was designed to replicate a state of relaxation, similar to an individual resting on a cushion. Assuming that the Von Mises stresses remain below the tensile strength of the PEEK material, it is expected that the implant will operate without any failure.

### 2.5. Fitting Accuracy 

**Figure 14:**
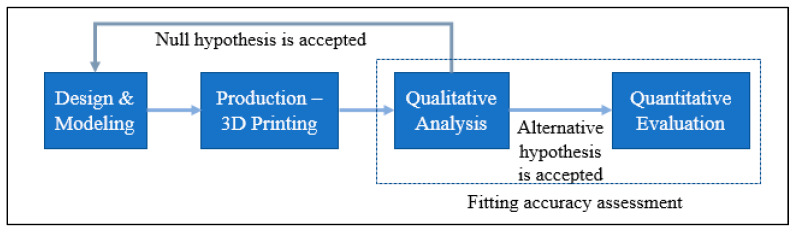
The methodology applied to facilitate accurate implant placement.

A PEEK-made cranial implant is positioned on a PLA-based skull for the purpose of evaluating the fitting correctness of the implant. Analyzing the fitting accuracy of the customized cranial implant must be an important research topic, given that it might dramatically change a patient’s overall appearance. According to Wyleżoł et al. [66], an initial visual evaluation of a manufactured implant shape by a team of professionals is required before subsequent processing and activities. This study, therefore, combines a quantitative assessment based on 3D scanning with a qualitative inspection relying on specialist experience. When conducting a qualitative assessment, a cranial implant is positioned on the skull and evaluated by experts using a visual analog score (VAS). A VAS is composed of ratings from 1 to 5, where 1 indicates a poor score, 2 is an acceptable score, 3 is a satisfactory score, 4 is a good score, and 5 is an excellent score [67]. Ten experts with backgrounds in medicine, surgery, and research are contacted for qualitative analysis. Each expert receives a PEEK implant and skull assembly on their own to evaluate the cosmetic and physiological performance of the implant considering elements like homogeneity, connection, and visual appeal. Five replicas of the implant are made and given to reviewers for anonymous evaluation in order to eliminate bias and assure reliability. After each expert gives a VAS to each implant replicate, the mean aesthetic score (MAS) is derived. The null and alternative hypotheses are investigated for statistically assessing the qualitative results [68,69]. The null hypothesis should be embraced when the MAS is less than or equal to 3 (H0: MAS ≤ 3). However, the alternative hypothesis prevails when the MAS is higher than 3 (Ha: MAS > 3) [70]. If the null hypothesis is not accepted following qualitative evaluation, the implant is evaluated quantitatively utilizing 3D scanning as shown in Figure 14. If the null hypothesis is true, the implant is modified and built again.

**Figure 15:**
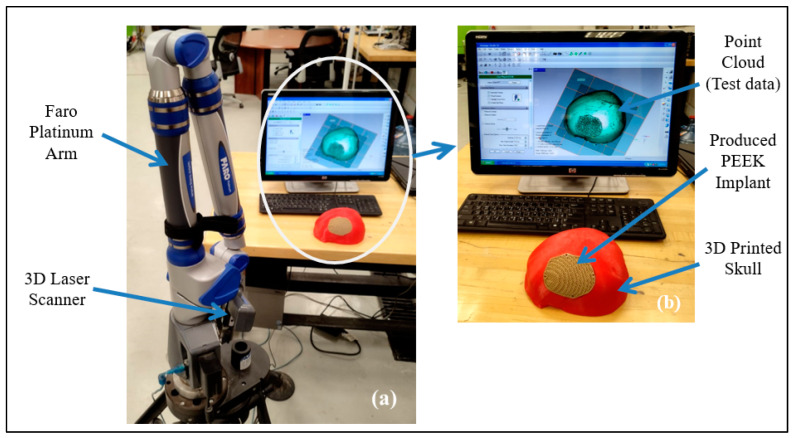
(**a**) 3D Scanning set up; (**b**) Gathered point cloud data.

After the qualitative analysis, the quantitative reassessment is crucial for determining the implant’s aesthetic and fitting success in terms of a metric. The quantitative evaluation involves calculating the implant’s deviance from the actual form of the skull. The implant-skull assembly is scanned utilizing scanning technology to obtain the point-cloud data in this procedure. The Standard Tessellation Language (STL) file, which is deployed as a test file against the reference file, is generated by further processing this point cloud data. As shown in Figure 15, the scanning in this work is accomplished with a laser scanner installed on a FARO Platinum arm (FARO, Lake Mary, FL, USA) (a). A technique named 3D comparison is used to match the test data (once it has been gathered through 3D scanning) to the reference skull, which represents the real shape. A 3D comparison is conducted using Geomagics Control (3D Systems, Rock Hill, SC, USA) in order to graphically assess the surface shifts between the investigating and the reference surfaces [71,72].

There are essentially three steps that must be completed in 3D comparison. The designation of objects is the initial step in the process since it notifies the software which surface is being studied (test surface) and which one is being used as a reference. The exterior of the implant-skull assembly is scanned and loaded as an STL file in Geomagics Control since the customized cranial implant is constructed based on the outside curvature of the skull. A digital replica of the patient’s actual (or clean) skull is employed as a reference to calculate the fitting accuracy of the reconstructed skull (secured with the fabricated implant). Aligning the test surface with the reference model is the second step. The test and reference objects are placed in the identical coordinate system using the best-alignment tool to accomplish this step. Eventually, deviation analysis utilizing 3D comparison is done in the third stage. The implant’s fitting accuracy is evaluated by determining the average deviation in a positive direction. The average deviation statistic is used because it shows a mean deviation in an outward direction, estimating the distance between the reconfigured skull (or the customized implant) and the natural cranium. The 3D comparison is applied to estimate the inaccuracy or deviation between the 3D Printed tailored implant positioned on the skull (restored skull) and the clean or real skull of the participant. This quantifies the overall fitting accuracy of the implant.

## 3. Results and Discussion

In this section, the biomechanical results and the implant fitting analysis are presented.

### 3.1. Biomechanical Results

**Figure 16:**
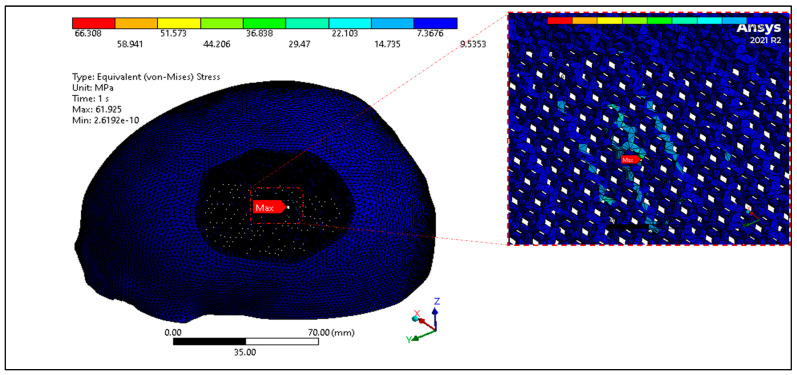
Equivalent Von mises Stress distribution of PEEK porous implant.

**Figure 17:**
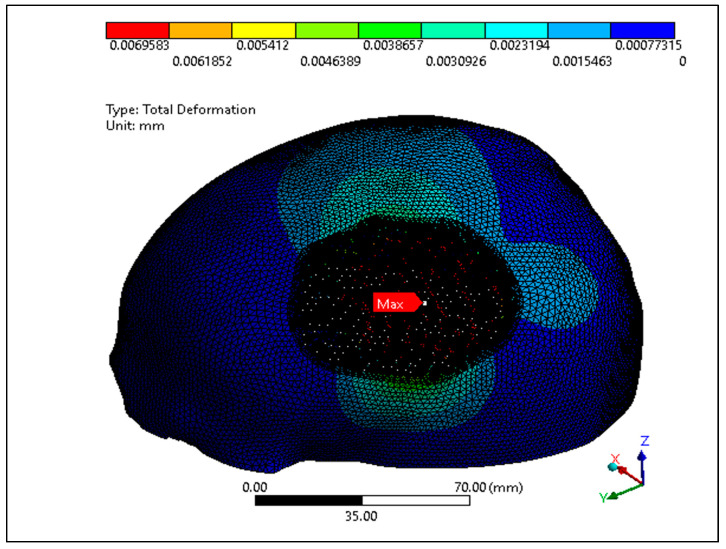
Total deformation of the PEEK porous implant model.

In this study, the porous PEEK implant is subjected to numerical simulation under a force of 50 N load using Ansys simulation software. The finite element analysis results as shown in Figure 16 reveals that the maximum von mises stress on the PEEK porous model is found to be 61.92 MPa which is very low and within the maximum yield and tensile strength of PEEK material (90/99.9) MPa. Figure 17 provides the view of the total deformation for the porous implant. As predicted, the maximum deformation was observed at the loading position. However, the magnitude was quite low, which was around 6 microns, and it went down to almost zero at the sites where the fasteners were attached. Eventually over the course of time, the tissue ingrowth and cell proliferation across the bone contact reinforce and stabilize porous implants.

According to the findings of several research studies, the porous implants promotes tissue ingrowth and cell proliferation across the bone interface over time, which increases the implant’s strength and stability [22,73]. Clinical studies suggest that the porous PEEK implants fully fuse with the use of peripheral skull tissue, and effectively promote the regeneration of new bone over a period of 6 weeks [74]. Based on the biomechanical results, we conclude the porous PEEK cranial implant can withstand a normal force involved in daily human activities and the implant would remain stable under typical loading conditions over a period of time. Li et al. [75] in his clinical study demonstrated that a small amount of new immature bone tissue integrates with the periphery of porous implant and after 12 weeks the pores are largely filled and new bone is formed and successfully bridged the bone defect.

### 3.2. Implant Fitting Analysis Results

The MAS for ten specialists across five replicates is obtained during the qualitative evaluation of the implant fitting. The hypothesis testing is carried out using a one-sample *t*-test in Minitab Statistical Software (Minitab 21, MINITAB Ltd., Coventry, UK). One-sample *t*-test is a statistical hypothesis test employed to ascertain whether a given population mean differs from a given value [76].

**Figure 18:**
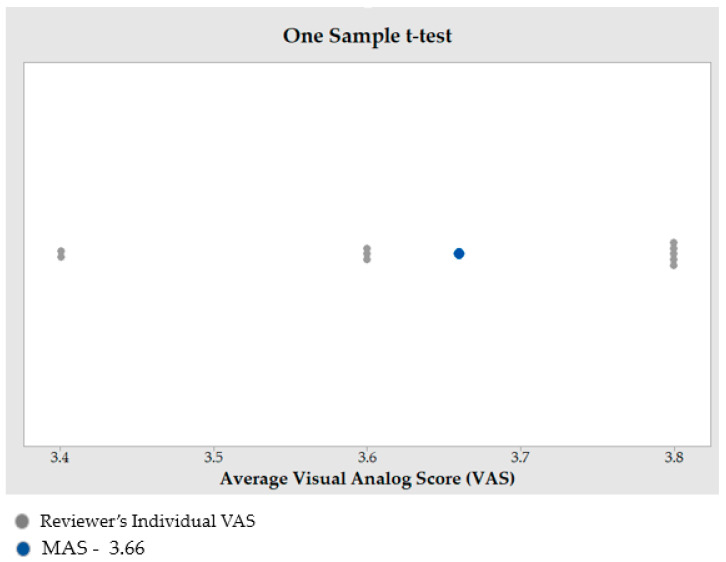
Outcome of one sample *t*-test to estimate the MAS.

When the *p*-value is lower than 0.05, the finding is regarded as statistically significant. In this analysis, the *p*-value is smaller than the significance level (α) of 0.05, rejecting the null hypothesis. The reconstructed skull has good aesthetics, as evidenced by the MAS of 3.66 out of 5 (*n* = 10) from this qualitative analysis (see Figure 18). Furthermore, a MAS greater than 3, indicates expert satisfaction and belief. This highlights the assertion that the implant design fits the skull well and has a good aesthetic appeal.

**Figure 19:**
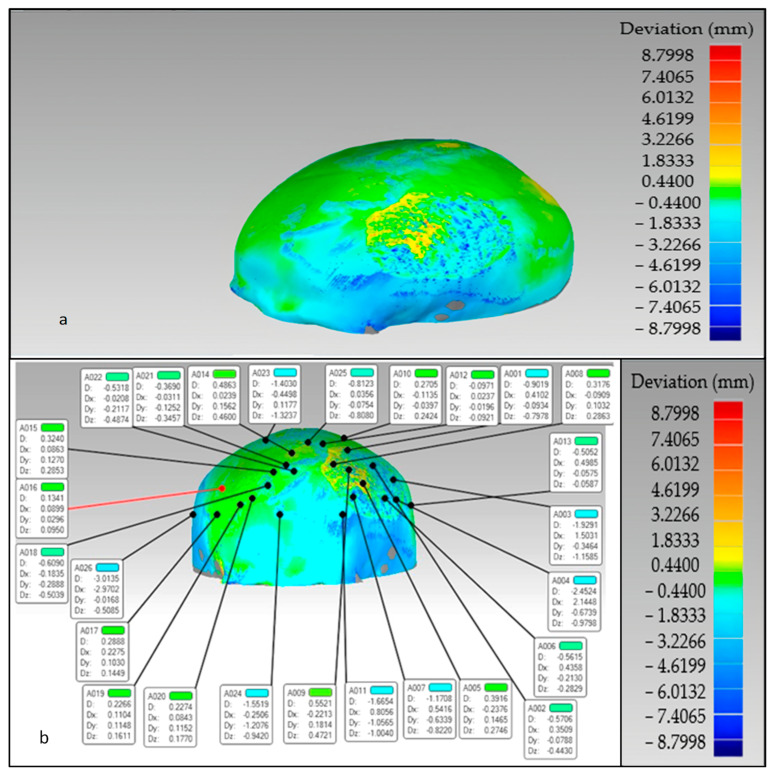
The overall divergence between the real skull (reference model) and reconstructed skull (**a**) overall error in the outer direction (**b**) error in the region of interest.

**Figure 20:**
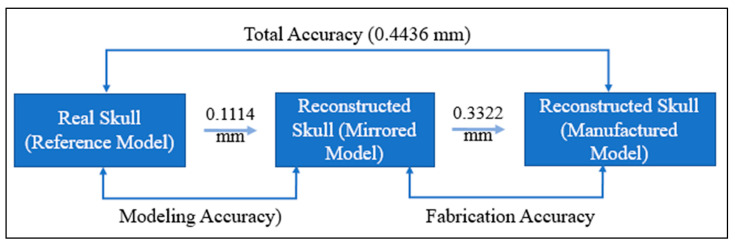
Total fitting accuracy of the porous PEEK implant in the reconstructed skull.

The findings of the 3D comparison analysis for the clean skull (reference model) and the reconstructed skull (skull with produced physical implant), are summarized in Figure 19a,b. The average divergence in the outside direction between the reference model (clean skull) and the reconstructed skull is 0.4436 mm which is less than 0.5 as reported by other researchers [77]. As seen in Figure 20, the total accuracy value actually combines the accuracy of the manufacturing process and the modeling approach, which in this case is mirror reconstruction. The accuracy of the mirror reconstruction in this investigation is approximately 0.1114 mm, whereas the accuracy of production is about 0.3322 mm. Additionally, the variation at the area of interest, between a cranial implant on the left side, and the right healthy side is just 0.0271 mm, which is very minimal assuming the complex anatomy of the vicinity.

**Figure 21:**
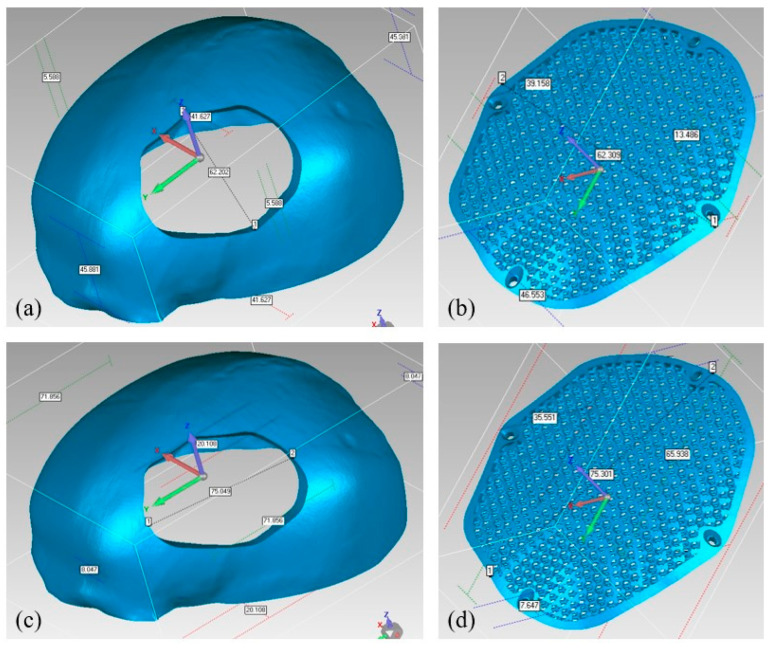
Gap Analysis (**a**) Cavity width in X-direction; (**b**) Implant width in X-direction; (**c**) Cavity length in Y-direction; (**d**) Implant length in Y-direction.

The reconstructed cranial implant made from PEEK is therefore appropriate and aesthetically appealing. In addition to the deviation analysis, Figure 21 displays the results of the gap analysis conducted in this study. The gap analysis reveals that the distance between the implant and the skull in both the X and Y dimensions is relatively modest. For instance, the typical implant length in the X direction is 62.25 mm and in the Y direction, it measures 75.03 mm. The average length of the (defect-free) cavity in the X and Y directions is 62.20 mm and 75.03 mm, respectively. It indicates a superior fit because the X and Y vector gaps are less than 0.06 mm (0.57 mm and 0.007 mm, respectively).

According to the findings of the aforementioned analysis results, the repaired skull is satisfactory and provides appropriate aesthetics and appeal. This investigation is one of the few attempts to analyze the correctness of the implant’s fitting. There hasn’t been much investigation into the fitting precision of medical implants in the earlier studies. To assure the credibility of the results, methodologies based on quantitative and qualitative ideas are applied. It is customary to have an experienced panel examine the quality of the implant from the outset. Following expert approval of the design and quality, the implants are fabricated and statistically tested using 3D scanning. The fabricated cranial implant scaffold, in addition to facilitating bone-implant ingrowth, also aides in reducing implant weight and assuring implant stability and long-term efficacy. The methodology used in this study helps the patient to achieve the desired esthetic outcomes while avoiding financial burden, mental tiredness, and the need for surgical adjustments.

## 4. Conclusions

PEEK has emerged as a promising substitute for metallic biomaterials in medical applications. To demonstrate its cranioplasty potential, a porous diamond scaffold PEEK implant with a pore diameter of 1350 μm and with a porosity of 70% is developed and fabricated utilizing FFF. The produced porous PEEK implant is subjected to numerical simulation and implant fitting analysis to quantify the design and fitting accuracy. The biomechanical investigation revealed that the head-pillow contact surface on the PEEK implant experienced a maximum stress of roughly 61.92 MPa when subjected to a load of 50N, which is within the limit of material’s tensile strength. The maximum level of deformation was observed to be notably lower, measuring approximately 6 microns. This finding indicates that the skull-implant structure can support the head’s weight under normal conditions. In addition, the implant fits the skull model accurately with a minimum variation of less than 0.4436 mm. This ensures that the PEEK porous cranial implant is extremely sturdy and reliable, thereby enhancing the implant’s capacity to withstand loads and ensuring safety during loading conditions. The study proves that PEEK porous implants could be a promising replacement for orthopedic and cranial procedures. In the future, subsequent research endeavors will be prioritized in analyzing porous PEEK implants in a clinical context.

## Figures and Tables

**Table 1:** Setting parameters for the Intamsys Funmat HT 3D printer.

Description	3D Printer Settings
Printing Technology	FFF
Extruder	Single
Extruder diameter (mm)	0.4
Layer thickness (mm)	0.15
Print speed (mm/s)	50
Print Speed (mm/s)	60
Slicing software	IntamSuite 3.6.2
Filament diameter (mm)	1.75
Build adhesion type	Raft

**Table 2:** The material parameters assigned to the finite element model [59,60].

Materials	Yield Strength (MPa)	Young’s Modulus (MPa)	Poisson’s Ratio
PEEK Porous Implant	99.9	3738	0.4
Skull (Cortical bone)	122	13,700	0.3
Titanium Screws	930	120,000	0.3

**Table 3:** Mesh data of the finite element model.

Components	Elements	Nodes
Skull bone	280,092	57,717
PEEK porous implant	508,440	107,020
Titanium screw_1	6414	1526
Titanium screw_2	6432	1540
Titanium screw_3	6422	1530
Titanium screw_4	6780	1596
Total	814,580	170,929

## Data Availability

The data presented in this study are available in the article.
